# Photoluminescence Enhancement in Erbium Nanoparticles via Controlled Phase Transformation

**DOI:** 10.1002/smll.202511510

**Published:** 2026-03-12

**Authors:** B. Almohammed, D. Barba, E. Haddad, F. Rosei, Fiorenzo Vetrone

**Affiliations:** ^1^ Centre Énergie Matériaux Télécommunications Institut National de la Recherche Scientifique Université du Québec Varennes Québec Canada; ^2^ Centre Énergie Matériaux Télécommunications Institut National de la Recherche Scientifique Campus Laval Université du Québec Laval Québec Canada; ^3^ MPB Communications Inc Pointe‐Claire Québec Canada; ^4^ Department of Chemical and Pharmaceutical Sciences University of Trieste Trieste Italy

**Keywords:** annealing, erbium oxide, erbium, laser ablation, nanoparticles, photoluminescence

## Abstract

Erbium‐based nanoparticles (Er‐NPs) were synthesized by Pulse Laser Ablation in Liquid (PLAL) and then heated at temperatures between 200°C–1000°C. A crystal structure transition from mixed cubic‐monoclinic phase to pure cubic Erbium oxide (Er_2_O_3_) phase is observed at 600°C, accompanied by strong volume compaction of the Er‐NPs. Through careful examination of their morphology, crystal structure, and chemical composition, we investigated the effects of post‐synthesis thermal annealing on the 4*f*–4*f* optical transitions associated with Erbium ions (Er^3+^). Our results indicate that thermal treatment conducted in a N_2_ atmosphere at ∼600°C promotes the stabilization of Er‐NPs in their favorable oxidation state for optimal red photoluminescence (PL) around 665 nm. This is connected to the thermo‐activated elimination of hydroxyl groups, the atomic densification that significantly reduces the Er‐NP size and crystal disorder, as well as the stabilization of oxygen ligands leading to cubic crystal symmetry. The contribution of several competing mechanisms to the observed PL is outpaced by energy transfer processes to the 4*f* emitting levels of Er^3+^, whose efficiency becomes optimal for reduced interatomic distances. The improved properties of Er‐NPs demonstrate their potential as next‐generation tunable nanomaterials for integration in optical sources, display devices, as well as in high‐reliability and temperature‐resistant thermal sensors.

## Graphical abstract

Er‐NPs synthesized by PLAL were thermally treated from 200°C to 1000°C. Annealing at 600°C produced a full transition to the cubic Er_2_O_3_ phase and removed non‐radiative surface species. This led to a fivefold increase in PL. The results show how oxidation and phase purity control the optical performance of Er‐NPs.

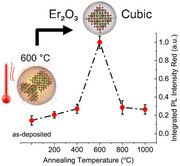

## 1 Introduction

Erbium oxide (Er_2_O_3_), a heavy rare‐earth compound, exhibits a range of attractive physical, chemical, and optical properties, making it valuable for diverse technological applications [1]. Its high dielectric constant (∼14) positions it as a strong candidate to replace SiO_2_ as a gate dielectric in metal‐oxide‐semiconductor devices [2]. The trivalent Er^3^^+^ ion, characterized by multiple metastable energy levels and long‐lived excited states, displays strong near‐infrared (NIR) absorption and efficient visible light emission, which are particularly beneficial for optoelectronic applications [3, 4, 5]. Previous work has shown that visible emission from the 4*f* shell of Er^3^^+^ ions are activated through the spatial and energetic redistribution of charge carriers, a process which is strongly influenced by the formation of Er─O bonds [6, 7]. This behavior is associated with the concept of an “oxygen cage,” wherein a well‐ordered coordination of oxygen atoms around the Er^3+^ ion facilitates the activation of optical 4*f*–4*f* transitions [8, 9]. The number and geometry of surrounding oxygen ligands play a crucial role in determining the PL efficiency of rare‐earth ions [10].

In Er_2_O_3_, Er^3^^+^ ions are an integral part of the host lattice, where each cation is surrounded by oxygen anions forming the nearest coordination shell. The removal of oxygen atoms disrupts this coordination, breaking the local symmetry and introducing asymmetric distortions around the metal ions [11, 12]. Defects such as oxygen vacancies, interstitials, and dopants create localized perturbations in the crystal lattice, leading to overall symmetry breaking. In metal oxides, such defect‐induced disorder can significantly alter both structural and electronic properties [13]. These disruptions influence the crystal field experienced by the 4*f* electrons of Er^3^^+^ ions, promoting non‐radiative energy transfer processes. Consequently, increased lattice distortion and point defect concentration are often correlated with reduced PL efficiency [14, 15, 16].

Er^3^^+^ ions exhibit a wide range of electronic transitions spanning from the ultraviolet (UV) to the NIR region. Moreover, Er^3^^+^ ions highly valuable for telecommunications and other photonic applications [17, 18]. Beyond photonics, Er_2_O_3_ has also been explored as a robust material for advanced coatings serving as tritium diffusion barriers, corrosion‐resistant insulators, and radiation‐tolerant layers in nuclear fusion environments [17, 18, 19, 20]. Furthermore, Er‐doped systems are widely used in fiber optics, optoelectronic devices, integrated photonic circuits, and lasers [21, 22, 23]. The technical implementation of Er‐NPs into various advanced systems may often require exposure to elevated temperatures during fabrication processes such as film deposition, thermal treatments, steaming or fiber drawing. These processes often involve heating in the range of less than 100°C to above 1000°C to ensure proper crystallization, adhesion, or dopant activation [24, 25].

The controlled synthesis of metal oxide nanoparticles (NPs) with tunable shape, size, and crystalline phase remains a central objective in materials science [26, 27, 28]. Among the available fabrication techniques, PLAL is considered promising due to its versatility, efficiency, and environmentally friendly nature [29, 30, 31, 32]. PLAL enables the growth of high‐purity, surfactant‐free NPs across a broad spectrum of elements [31]. The synthesis protocol for Er‐NPs used in this study has been described in previous work [33].

Since the structural and chemical state of materials critically influence their magnetic, electrical, optical, and thermal properties, it is essential to understand their crystal phases [28]. Er_2_O_3_ typically crystallizes in three polymorphs: cubic, monoclinic, and hexagonal. In this study, we refer to the cubic and monoclinic phases as C and M, respectively. Among these, the cubic phase is the most thermodynamically stable under ambient pressure over a wide temperature range, while the monoclinic and hexagonal phases are metastable. The metastable phases are scientifically interesting because they exhibit different optical and structural properties compared to the cubic phase, offering potential advantages for specific applications [34, 35].

One of the primary challenges in achieving high‐efficiency luminescent Er‐NPs consists in mitigating non‐radiative losses caused by structural and surface‐related defects [36, 37]. Hydroxyl surface contamination is commonly cited as a quenching mechanism typically requiring annealing for effective removal [36]. In previous work, we showed that the dominant factor responsible for PL quenching is not hydroxyl groups only, but rather the formation of pure Er nanoclusters [33]. Such clusters arise due to oxygen desorption during high‐temperature treatment, prompting increased Er─Er interactions that facilitate non‐radiative energy transfer.

To address this issue, thermal annealing under controlled conditions is essential, both for eliminating residual hydroxyls and carbon species introduced during synthesis as well as for enhancing the structural stability and optical performance of the NPs. While prior studies have primarily focused on improving NPs dispersion and minimizing agglomeration [38, 39], relatively few have explored the influence of annealing temperature on the crystal structure and chemical state factors that are crucial for optically activating Er^3^^+^ ions.

Here we aim to fill that gap by investigating the impact of post‐synthesis thermal annealing on the physicochemical and PL properties of Er‐NPs. The structural evolution of the NPs was examined using X‐ray diffraction (XRD), scanning electron microscopy (SEM), and transmission electron microscopy (TEM). X‐ray photoelectron spectroscopy (XPS) was employed to probe changes in surface chemistry and oxidation states, with additional depth profiling enabled by ion etching. PL spectroscopy was used to assess the PL response of the Er‐NPs as a function of annealing temperature. Our results demonstrate that annealing profoundly alters the optical behavior of Er‐NPs, with significant improvements in PL emission linked to the removal of hydroxyl groups and the stabilization of Er^3^^+^ ions in favorable oxidation states. Our results highlight the key role of oxygen coordination in activating 4*f* electronic transitions and provides valuable insights for optimizing Er‐NPs for photonic applications.

## 2 Results and Discussion

### 2.1 Structure and Crystalline Phase Analysis

**FIGURE 1:**
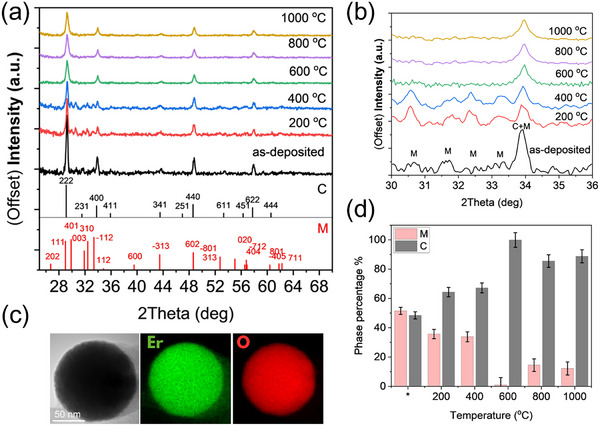
(a) X‐ray diffraction (XRD) patterns of Er‐NPs as‐deposited and annealed at various temperatures (200°C, 400°C, 600°C, 800°C, and 1000°C). The reference patterns for cubic Er_2_O_3_ (ICDD PDF‐4 No. 01‐077‐0459, black lines labeled “C”) and monoclinic Er_2_O_3_ (ICDD PDF‐4 No. 04‐001‐8737, red lines labeled “M”), (b) Magnified XRD region (30°–36° 2θ), (c) TEM and EDS mapping of as‐deposited Er‐NPs (d) the percentage of cubic (C) and monoclinic (M) phases as a function of annealing temperature, as determined by Rietveld refinement of the XRD patterns. The asterisk (^*^) refers to the as‐deposited sample (before annealing).

The XRD patterns of the as‐deposited and annealed samples are shown in Figure 1a. For as‐deposited reference samples, impurity and mixed crystalline phases are observed. Compared to the standard PDF cards, it is found that the structure of these Er‐NPs is a mixture between the cubic Er_2_O_3_ (ICDD 01‐077‐0459) [40] and monoclinic Er_2_O_3_ (ICDD 04‐001‐8737) [33, 41] phases. Using the Rietveld [42] method for determining the relative concentration of each phase, the as‐deposited sample is found to be monoclinic at 66% and cubic at 34%.

Once the samples are annealed, the diffraction peaks related to the monoclinic Er_2_O_3_ phase begin to diminish. Specifically, the monoclinic peaks at 30.6° (−401), 31.8° (003), 32.4° (310), 33.3° (−112), and 34.6° (112) start to disappear and are no longer present at 600°C, as illustrated in Figure 1b. Concurrently, the diffraction peak at 34° (400) shifts toward the cubic Er_2_O_3_ position, indicating progressive phase transformation from monoclinic to cubic structure. This result indicates that for this range in temperature, the crystalline phase of Er_2_O_3_ changes from the mixed monoclinic and cubic phases to the almost pure (∼97%) single cubic phase. Figure 1d shows the evolution of the crystalline phase transitions with increasing annealing temperature, as determined by the Rietveld refinement method.

This observation is particularly important because 600°C is identified as the optimal annealing temperature to maximize PL efficiency, achieve the complete crystalline transition of Er‐NPs to the cubic Er_2_O_3_ phase, and minimize non‐radiative losses (as discussed in section 2.5). This finding is in agreement with previously published work [34, 43], where heat treatments conducted at 700°C for 3 h were shown to induce crystalline phase transitions in Er_2_O_3_ and erbium oxyfluoride. The difference in the thermal annealing durations, as well as the conditions of the annealing reported in previous parametric studies, namely in vacuum for [39] vs. thermal treatment in atmosphere pressure under N_2_ flux here. Such variations in annealing environments are expected to influence the kinetics of phase evolution, thereby shifting the temperature at which a complete crystalline phase transition occurs reported at 700°C in earlier studies compared to 600°C in this work.

### 2.2 Size and Morphology of Er‐NPs

**FIGURE 2:**
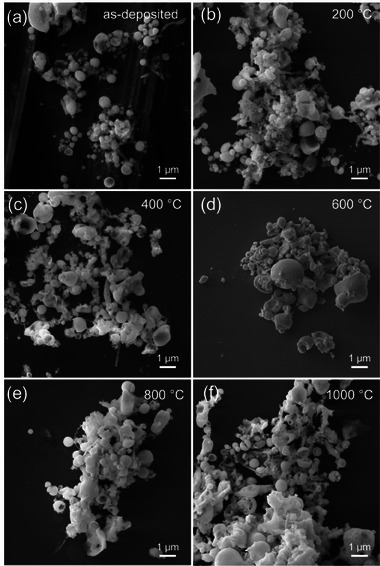
The SEM images of the Er‐NPs (a) as‐deposited, (b) 200°C, (c) 400°C, (d) 600°C, (e) 800°C, and (f) 1000°C.

**FIGURE 3:**
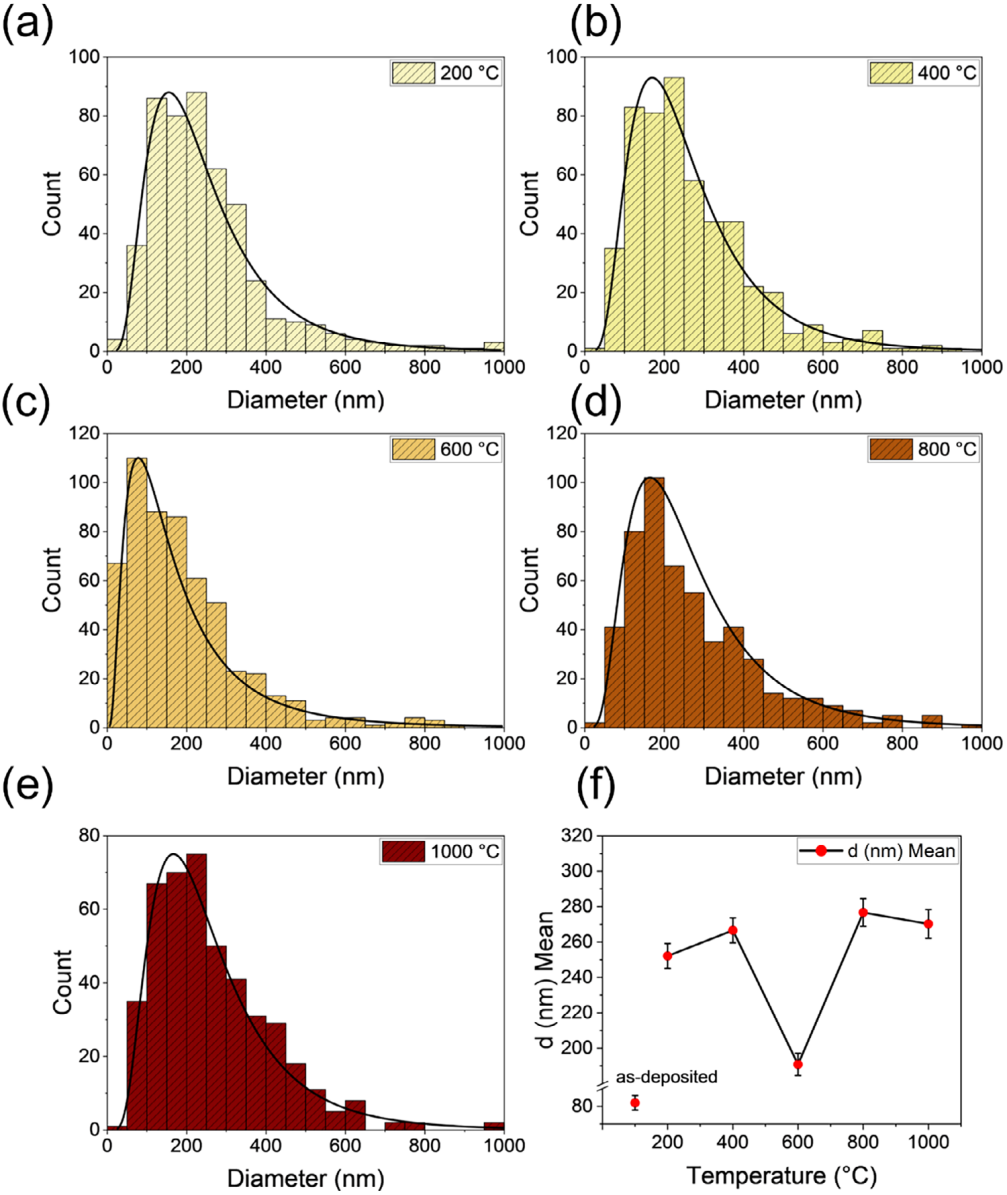
The size‐distribution of Er‐NPs at different annealing temperatures: (a) 200°C, (b) 400°C, (c) 600°C, (d) 800°C, (e) 1000°C, and (f) the mean NP size as a function of annealing temperature, including the as‐deposited sample.

The examination of the Er‐NPs' shape through SEM images shown in Figure 2a–f indicates that the specimens are composed of aggregated nanospheres with similar spherical morphology. In the as‐deposited sample, these objects consist of nanospheres (average diameters of 80 nm). The size of the NPs is defined as the peak (mean) diameter of the size distribution, obtained by fitting the measured particle size histogram with a log‐normal function, as shown in Figure 3a–e. The figure demonstrates the general increase in NPs size with rising annealing temperatures, as shown by the mean diameters of 252 ± 16 nm, 266 ± 18 nm, 190 ± 9 nm, 276 ± 20 nm, and 270 ± 17 nm, measured for Er‐NPs annealed at 200°C, 400°C, 600°C, 800°C, and 1000°C, respectively. Such changes in size mainly result from oxidation, usually leading to volume expansion by 20%, followed by densification arising from crystal reordering and atom outgassing before and after heating. During the crystalline phase transition from a mixed monoclinic‐cubic structure to a pure cubic structure by annealing at 600°C, volumetric expansions due to lattice mismatches, voids and vacancies, stacking defects, and point defects are significantly reduced. This effect allows for optimal compaction of single‐crystal Er‐NPs as evidenced by the smaller diameter of the Er‐NPs observed in this sample.

The increase in NP size observed beyond 600°C can be attributed to thermally activated processes that occur during annealing, including the breakdown of Er─O, oxygen release, and surface hydroxyl bonds, as supported by the tabulated data [44, 45, 46], and subsequent structural reorganization (Figure 1d). The release of oxygen during the annealing process may lead to a reduction in the oxide layer or an increase in the internal of the NPs, which can drive a rearrangement of the material. These transformations promote the formation and growth of inner nanoclusters and may lead to densification or recrystallization of the NPs, as thermal energy overcomes diffusion and bonding barriers [6]. Additionally, particle growth mechanisms become more efficient at elevated temperatures because of the sintering and coalescence of small Er‐NPs, as well as Ostwald ripening, where larger particles grow at the expense of smaller ones. All these effects are cumulative, contributing to the observed increase in the average size of NPs [47, 48].

In Figure 3f, the mean size of the NPs is shown as a function of the annealing temperature. The reduction in particle size observed at 600°C, followed by an increase at 800°C and 1000°C, suggests the occurrence of decomposition, sintering, and possible structural reorganization of Er‐NPs during annealing. The non‐monotonic variation in particle size with temperature reflects the competing effects of these thermal and structural processes. The observed behavior represents a balance between densification, surface reconstruction, and phase stabilization, consistent with crystal transitions between cubic and monoclinic phases. The reported changes in the bonding and chemical compositions, which also influence the particle size, will be investigated by XPS and thermogravimetric analyses (TGA) in the next sections.

EDS measurements were performed on as‐deposited Er‐NPs as illustrated in Figure 1c. The compositional analysis of the nanospheres confirms the presence of erbium (Er) and oxygen (O), visualized in Figure 1c in green and red, respectively. The elemental mapping over a single isolated NPs in the TEM image reveals a uniform spatial distribution of both elements across the entire particle. This apparent homogeneity suggests that the as‐deposited Er‐NPs are predominantly oxidized within the region probed by the electron beam. The observed oxidation is likely due to chemical interactions between sputtered Er atoms and dissolved O in water during the PLAL synthesis process.

### 2.3 Thermo‐Gravimetric Analysis (TGA)

**FIGURE 4:**
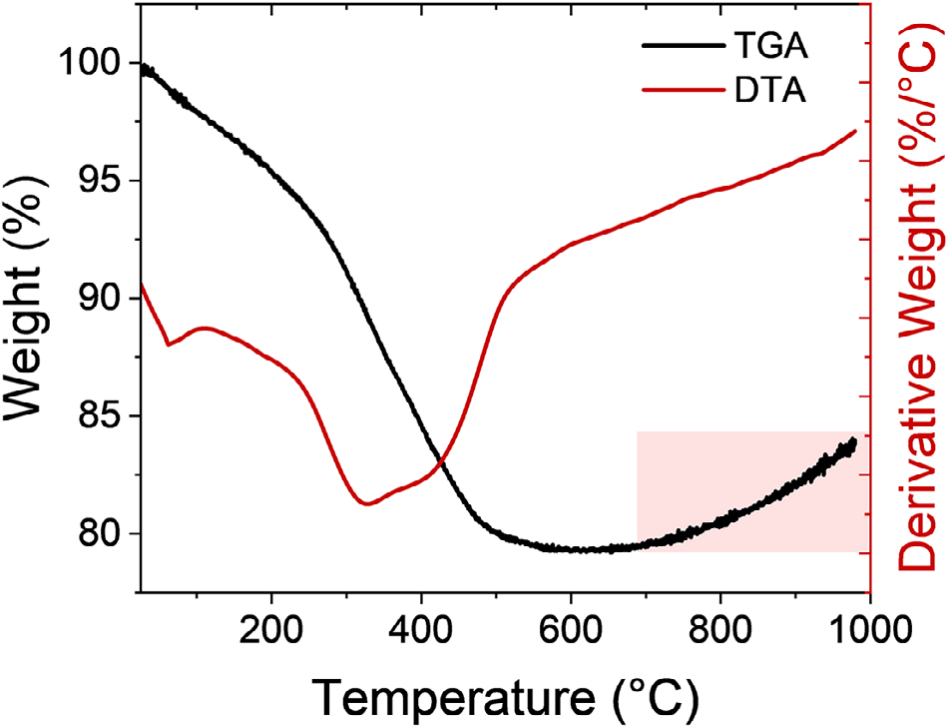
TGA/DTA thermograms of Er‐NPs recorded under argon atmosphere.

Thermogravimetric and differential thermal analysis (TGA/DTA) were used to monitor weight loss of the Er‐NPs during annealing, as shown in Figure 4. According to the TGA results, a total weight loss of ∼23% occurs at different stages. At the beginning of the annealing process, the slight weight loss under 100°C can be attributed to the evaporation of H_2_O in the sample [49], demonstrating that the 10 min baking at 150°C is necessary and effective to solidify the sample. In the second step, from 125°C to 327°C, the weight loss of ∼16% comes from the dehydroxylation of the NPs, structural rearrangement processes, and due to the carbon burning [44, 49]. When the annealing temperature increases to 560°C, a weight loss of ∼3% occurs, which is associated with the decomposition of the Er_2_O_3_ in the samples. An exothermic event is observed in the DTA curve at ∼600°C, corresponding to the phase transformation and crystallization into the stable cubic Er_2_O_3_ structure [50]. Furthermore, no significant weight loss was observed at higher temperatures, meaning that the complete decomposition of cubic Er_2_O_3_ and the densification of the Er_2_O_3_ metal‐oxide bonding. The weight gain above ∼750°C is possibly due to the rearrangement processes [51, 52]. This behavior is consistent with the formation of metallic Er‐NPs, as the emergence of Er─Er bonds being stronger than Er─O bonds may indicate a reduction in the oxide content and the stabilization of Er‐rich phases under high‐temperature conditions.

Based on the TGA/DTA results, the heat‐treatment temperatures confirm the structural rearrangement of the Er‐NPs, as observed in the XRD patterns. The stable cubic Er_2_O_3_ phase forms at ∼600°C [23, 53], with this transformation accompanied by distinct thermal events including dehydration and dehydroxylation processes.

### 2.4 Chemical Composition Analysis

**FIGURE 5:**
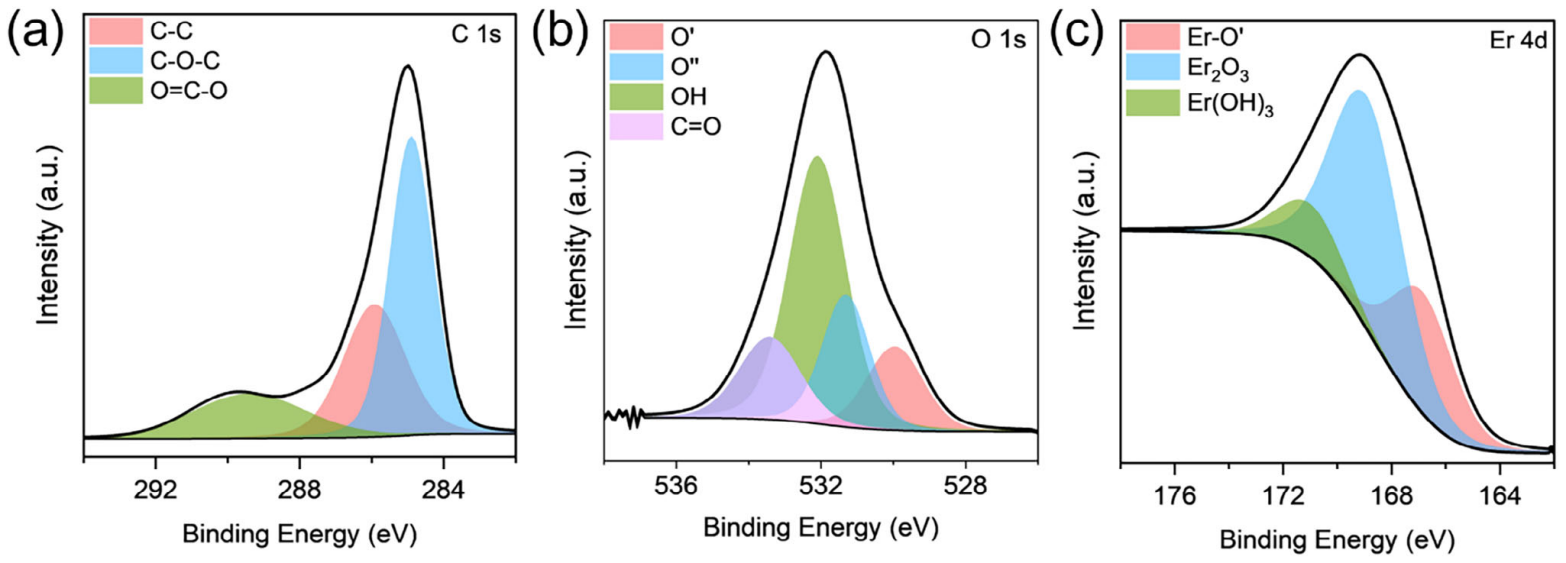
XPS analysis of Er‐NPs as‐deposited (a) deconvoluted C 1s spectrum, (b) deconvoluted O 1s spectrum, (c) deconvoluted Er 4d spectrum.

Er‐NPs were subjected to thermal treatment to determine the threshold temperature at which hydroxides decompose, leading to the formation of a stable oxide phase. This process was subsequently analyzed using XPS to provide a comprehensive understanding into the chemical composition and phase stability of the annealed samples. In previous work, we conducted an in‐depth XPS analysis of Er‐NPs, providing detailed insights into their surface chemistry [33]. For the deconvolution of the high‐resolution XPS spectra, an advanced fitting procedure was employed to accurately resolve the chemical states present. For a more detailed explanation of the fitting methodology and spectral interpretation, we refer to the detailed procedures outlined in Refs. [33, 54, 55]. The deconvoluted C 1s spectrum of Er‐NPs reveals three prominent peaks at binding energies of 284.9, 285.9, and 289.4 eV, which are attributed to the presence of C─C, C─O─C, and O═C─O functional groups, respectively, in Figure 5a. Moreover, the high‐resolution XPS peak related to O 1s binding energy is shown in Figure 5b. The double‐peaks at 529.0 and 531.1 eV are assigned to oxygen from the Er─O’ and Er_2_O_3_, respectively [56, 57, 58]. The peak observed at 533.5 eV is assigned to carboxylic C═O bonds and contributions from the substrate, whereas the signal at 532.0 eV corresponds to O─H groups resulting from the interaction between Er‐NPs and water molecules present in the colloidal solution [7, 54]. In Figure 5c, the Er 4d spectral region is deconvoluted into three distinct components corresponding to Er─O’ at 167.4 eV, Er_2_O_3_ at 168.0 eV, and Er(OH)_3_ at 170.1 eV [59]. To determine these values, the energy separation between peaks was constrained to a maximum variation of 0.5 eV, while allowing spectral shifts of up to 1 eV in peak positions. This flexibility accounts for potential variations arising from nanoscale dimensional changes, differences in the chemical environment, and instrumental factors. During the fitting procedure, a standard mixed Lorentzian‐Gaussian line shape function (LA 1.64) was used to model the XPS peaks of all Er‐based components [60]. All the deconvoluted XPS spectra are presented in the Figure S1 of the supplementary information (SI) section, showing the evolution of the Er 4d and O 1s chemical states as a function of the annealing temperature.

**FIGURE 6:**
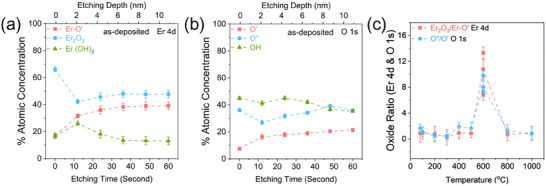
Depth‐dependent atomic concentration profiles for the as‐deposited Er‐NPs as a function of ion etching time of (a) Er 4d components: Er─O′, Er_2_O_3_, and Er(OH)_3_, (b) O 1s components: O′, O″, and OH. (c) Oxide ratios Er_2_O_3_/Er─O′ from Er 4d and O″/O′ from O 1s as a function of annealing temperature.

The surface chemical composition and depth profiles of the as‐deposited and thermally annealed Er‐NPs were analyzed using XPS. XPS Spectra were used to extract elemental atomic concentration as a function of etching time and temperature (Figure 6). To evaluate the depth‐dependent chemical changes induced by annealing, ion beam etching was performed using a 3 kV Ar^+^ ion beam, tilted at 45° relative to the sample surface. Each etching step lasted 12 s, with a beam current of 0.7 µA directed onto a sample area of 3.3 × 2.8 mm^2^.

From the measured beam current, an ion flux density of 5.7 × 10^1^ ^4^ Ar^+^/cm^2^ was calculated. The molecular concentration of the target was assumed to be 1.4 × 10^2^ ^2^ mol/cm^3^, and an average sputtering yield of 4.2 target atoms per incident Ar^+^ ion was used. This yield was estimated using SRIM‐TRIM Monte Carlo simulations [61] under the specified etching conditions (3 kV acceleration voltage and 45° incidence angle). Following a similar methodology to that used for analyzing colloidal core‐shell systems exposed to proton fluxes [62], the sputtering process was found to release approximately 3.1 Er atoms and 5.4 O atoms per incoming Ar^+^ ion.

Assuming that the ejection of an Er or O atom also results in the removal of any contaminant adsorbed on the surface, the calculated erosion rate at the external surface of the Er‐NPs was approximately 1.8 nm per 12 s etching cycle. This sputtering rate allows us to correlate the etching time with the thickness of material removed by the Ar^+^ sputtering beam. This scale is reported in nm, on the top horizontal axis of the Figure 6a,b.

Figure 6a shows the evolution of the spectral components Er 4d inside Er‐NPs, illustrating changes in the relative concentrations of Er─O′, Er_2_O_3_ and Er(OH)_3_ as the probe penetrates into the NPs. The corresponding atomic concentrations of the O 1s components (O′, O″, and OH) are reported on Figure 6b, as a function of the depth at which they have been measured subsequently to ion etching. Such XPS analyses were conducted in all annealed samples with the same procedure, as summarized in Figure S2a–f of the SI section, presenting the in‐depth evolution of the Er and O concentrations inside the annealed Er‐NPs. The initial etching steps reveal a noticeable decrease in surface contaminants, such as hydroxides and carbon‐bound oxygen, indicating their presence is largely limited to the outermost layers, resulting from post‐annealing in the ambient environment. Furthermore, the calculated oxide ratios Er_2_O_3_/Er─O′ from Er 4d and O″/O′ from O 1s as a function of annealing temperature are shown in Figure 6c. These ratios highlight the temperature‐dependent oxidation behavior. The trends in Figure 6c show that the Er_2_O_3_ is approximately 20% atomic concentration in the as‐deposited Er‐NPs. This initial concentration suggests that a significant portion of the oxygen in the system is associated with hydroxides or other surface‐bound species, rather than fully oxidized Er. Once the annealing temperature is increased, the atomic concentration of Er_2_O_3_ in the O 1s, and Er 4d peaks rises, reaching a maximum at 600°C, and then decreases for annealing at 800°C and 1000°C.

This result indicates that the Er‐NPs annealed at 600°C are those that contain the highest degree of Er oxidation. To ensure the reliability of this characteristic for the sample annealed at 600°C, the in‐depth chemical analysis was repeated several times, confirming the reproducibility of the observations and improving the accuracy of the oxidation state assessment at this critical annealing temperature.

However, with increasing annealing temperature, multiple reaction stages are triggered, leading to the release of gases and facilitating structural rearrangements. These processes likely contribute to the nucleation, growth, and internal reorganization of the Er‐NPs, as supported by the crystallographic changes observed in the XRD patterns [63, 64]. For elevated annealing temperature, the reorganization of Er‐NPs could lead to the formation of different bonds by overpassing specific chemical energy thresholds required to break these bonds [7, 59, 65]. At 600°C, significant oxidation takes place, resulting in the complete conversion to a stable Er_2_O_3_ phase [38, 39, 53, 66]. This is the critical range where the bulk of the hydroxide content is fully converted into oxide, as reflected by TGA.

Above 600°C, further heating leads to rearrangements within the NPs. Although oxidation is largely complete by 600°C, additional flow of thermal energy promotes densification of the oxide phase, leading to compaction and sintering of the particles. At this stage, outgassing of any remaining volatile species (such as oxygen) may occur, which can contribute to a slight reduction in Er_2_O_3_ content. The release of oxygen from the lattice may lead to localized defects or vacancies within the oxide structure. Additionally, at elevated temperatures, the system shows evidence of oxygen loss, which may result in the partial reduction of Er_2_O_3_ to a sub‐stoichiometric oxide (Er_2_O_3‐x_) or the formation of mixed phases [67, 68]. This temperature‐dependent oxidation behavior highlights the crucial role of thermal treatment in driving the complete oxidation and stabilization of the Er_2_O_3_ structure.

### 2.5 Activation of Er^3+^ in Er‐NPs

**FIGURE 7:**
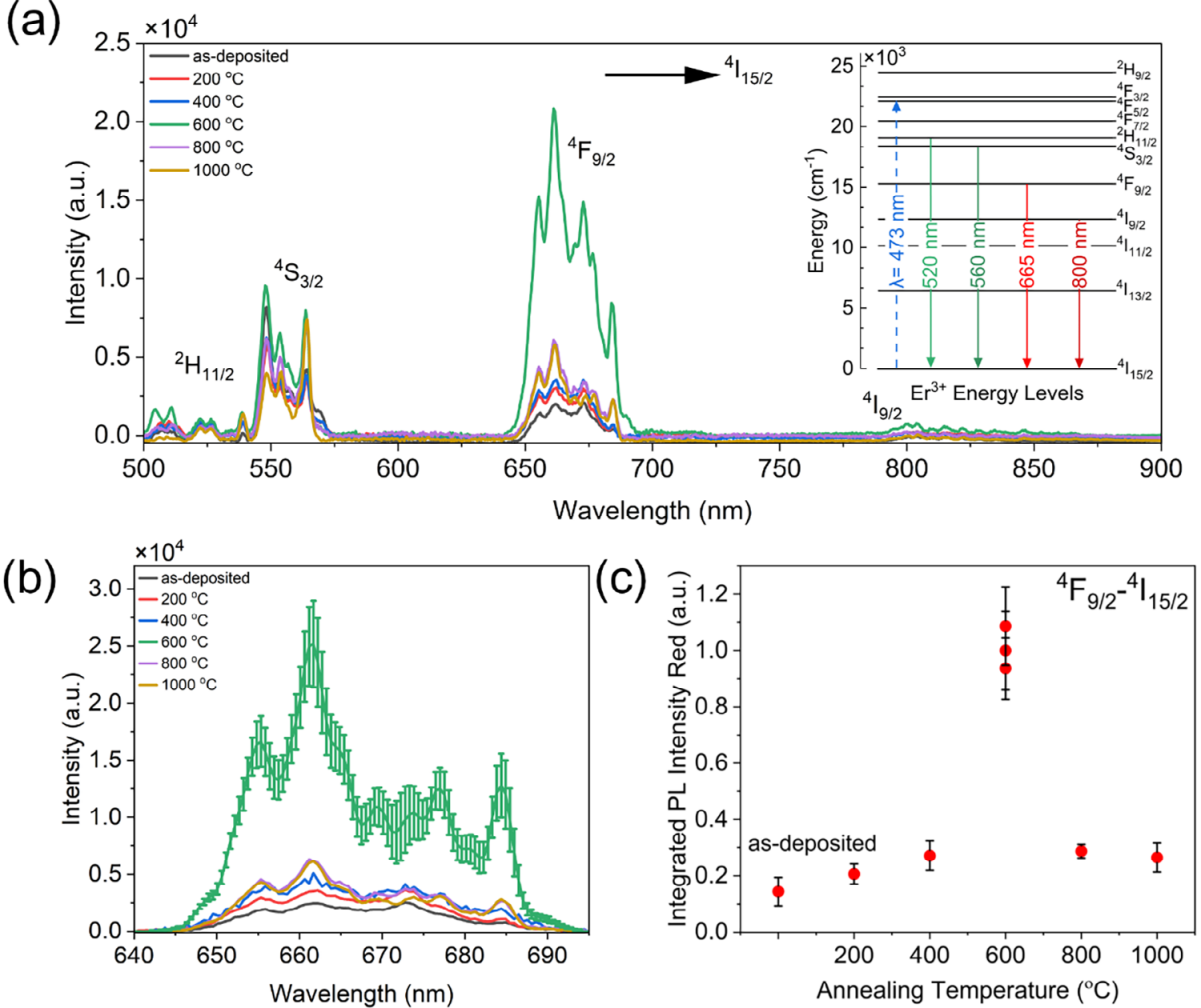
(a) PL emission spectra of Er‐NPs from 500 to 900 nm, highlighting transitions from excited states to the ground state, and Er^3+^ energy level (inset), (b) PL red emission at 665 nm for the ^4^F_9/2_ → ^4^I_15/2_ transition, (c) PL integrated intensities for red emission (error bars represent the standard deviation of three replicated experiments).

Figure 7a presents the room temperature PL spectra of Er‐NPs, recorded in the 500–900 nm range for both as‐deposited and annealed samples. The optical transitions involved in the sample PL are related to the excited energy levels of Er^3+^ ions, as shown with vertical arrows of different colors in the inset of Figure 7a. Three prominent spectral regions related to efficient Stark splitting are observed around 560 nm, 665 nm, and 800 nm. They correspond to the characteristic electronic transitions of Er^3+^ ions from the ^2^H_11/2_ and ^4^S_3/2_ states (near 520 and 560 nm), the ^4^F_9/2_ state (around 665 nm), and the ^4^I_9/2_ state (near 800 nm) down to the ^4^I_15/2_ ground state, respectively.

The optical signal around 665 nm, corresponding to the ^4^F_9/2_ → ^4^I_15/2_ transition, was isolated from overlapping spectral features and background contributions, as shown in Figure 7b. The error bars shown in Figure 7b correspond to the standard deviation calculated from the three repeated annealing experiments at 600°C. The integrated intensity of this peak was used to evaluate the impact of annealing on the optical properties of the NPs. Optical activation of the Er^3+^ ions was observed at all annealing temperatures, with a marked enhancement in PL intensity as the temperature increased. The emission reached its maximum at 600°C, beyond which it declined significantly, mainly due to oxygen desorption and outgassing [69, 70]. Figure 7c illustrates this trend by plotting the integrated PL intensities of as‐deposited and annealed samples (200°C–1000°C) as a function of annealing temperature.

The trend observed in Figure 7c shows that the PL emission from the as‐deposited sample is relatively weak when compared to the annealed samples. This low emission is expected, as low‐temperature drying leaves behind residual deionized water and surface hydroxyl groups, both of which promote non‐radiative recombination processes. As the annealing temperature increases, the PL signal improves significantly, reaching a maximum at 600°C. At this temperature, the optimal PL emission is approximately five times higher than that of the as‐deposited sample. The reliability of the sample for thermal annealing at 600°C was validated by three independent annealing experiments conducted at different intervals of a few weeks.

Such a temperature‐dependence of the PL intensity suggests the occurrence of competing chemical processes. Below 600°C, the progressive removal of surface hydroxyls and structural reorganization enhance the optical emission of Er^3^^+^ ions. However, above 600°C, the thermal energy becomes sufficient to break the Er─O bonds [69], resulting in oxygen desorption, outgassing and possible sintering, all of which can contribute to the reduction of PL emission [71, 72, 73]. This intensity lowering results from the substitution of Er─O bonds by Er─Er bonds during the annealing and/or the sample cooling down, which all act as quenching PL centers [33].

From a physical aspect, the PL is regulated by optical selection rules, related to the nature of the levels involved in electron transitions and the local crystal symmetry (parity rules), as well as the occupation of excited electron levels, which is connected with optical absorption and energy transfer toward the excited Er^3+^ emitting states. All these contributions are sensitive to local defects, which promote the thermalization of excited electrons through non‐radiative processes inside Er‐NPs. Due to the mixed crystal structure of Er‐NPs and the high concentration of defects found by XRD, we infer that these non‐radiative effects are dominant. Such a feature is consistent with the green (^4^S_3/2_ → ^4^I_15/2_) and red (^4^F_9/2_ → ^4^I_15/2_) PL where similar intensities are observed, which results from a highly efficient Stark separation and exhibit the spectral signature expected for low local symmetry [74, 75].

The 4*f–*4*f* transitions are electric dipole‐forbidden, meaning that only emissions from Er^3+^ occupying non‐inversion centers or inversion centers perturbed by local lattice distortions or defects (leading to asymmetry at longer ranges) are allowed. According to Judd‐Ofelt (JO) theory quantifying the strength of electric dipole transitions through the JO parameters Ω_t_ (*t* = 2, 4, 6) [76, 77], the intensity of the red PL (^4^F_9/2_ → ^4^I_15/2_) is proportional to Ω_2_. This latter should be greater for monoclinic Er_2_O_3_ having a lower symmetry than for cubic Er_2_O_3_. Consequently, if the variations in red PL intensities would be only (or mainly) mediated by variations in crystal symmetries, the PL from cubic Er‐NPs should be lower than that of a pure monoclinic or mixed monoclinic‐cubic crystallites. Such a feature is in complete contradiction with what is seen in this work, as well as in the previous investigation of Er^3+^ doped cubic and monoclinic rare earth sesquioxides [34, 78, 79], who also reported less intense red emission from Er^3+^ in mixed crystal phase than in pure cubic phase, in agreement with our result.

**FIGURE 8:**
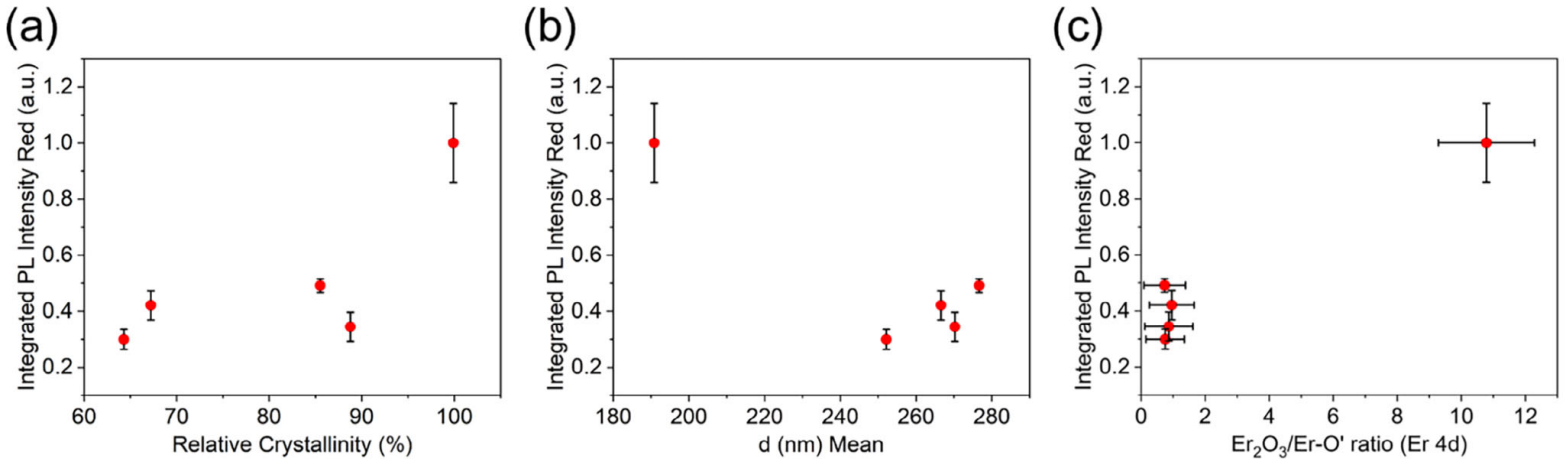
Integrated PL intensity of the red emission plotted as a function of (a) the relative crystallinity of the cubic phase, (b) the mean diameter of the Er‐NPs, and (c) the oxide ratio obtained from the Er_2_O_3_ / Er─O’ components of the Er 4d XPS spectra.

**FIGURE 9:**
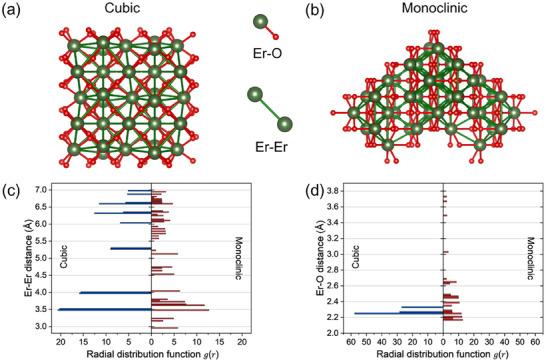
Schematic representation of Er─Er and Er─O distances in the layered sublattice: (a) Er_2_O_3_ Cubic and (b) Er_2_O_3_ Monoclinic. (c), and (d) Comparison of the distribution of Er─Er and Er─O distances between cubic and monoclinic, respectively. The radial distribution function *g(r)* gives the probability of finding another atom at a distance r from a reference atom.

In Figure 8, the normalized PL intensity of ^4^F_9/2_ → ^4^I_15/2_ transitions (Figure 7b) was plotted as a function of the cubic fraction of Er_2_O_3_ measured by XRD (Figure 8a), and the average diameter of Er‐NPs extracted from SEM analysis (Figure 8b). Although the PL may vary with the crystallinity and size of Er‐NPs (both reported in Figures 1d and 3f, respectively), no direct clear correlation can be established between the PL intensity and the crystalline cubic phase fraction, nor between the PL intensity and the average dimensions of Er‐NPs. Indeed, the PL intensity variations do not follow any continuous nor linear trend that could reveal their strong dependence on the crystal symmetry and the Er‐NPs’ size parameters. These remarks make the change in excitation energy transfer efficiency toward the emitting levels of Er^3+^ the most important contribution to the red PL intensity variations. These energy exchanges seem to be either connected to the number of Er^3+^ oxygen ligands (as well as to the atom spacing, see below Figure 9), as suggested from Figure 8c, showing the variation of PL intensity upon the concentration of Er─O’ measured by XPS. Here, the data indicate that the intensity of PL emission increases continuously and almost linearly with the fraction of oxidized Er present in Er‐NPs. In addition to its consistency with our previous study on Er‐NPs [33], this result reveals that the degree of Er oxidation appears to be the parameter that most influences the intensity of their PL emission.

The energy transfer between ions also highly depends on the spacing between adjacent atoms [34, 43]. The closer the Er─Er and Er─O distances, the higher the probability of energy transfer between them [34, 80]. In the cubic phase, Er_2_O_3_ adopts a body‐centered cubic structure with space group *Ia‐3*. The unit cell contains 16 formula units, amounting to 32 Er^3^^+^ ions and 48 O^2^^−^ anions. The Er^3^^+^ ions are distributed across two distinct crystallographic sites where 8 Er^3^^+^ ions occupy the b sites, which exhibit C_3i_ (inversion and threefold rotational) symmetry. The other 24 Er^3^^+^ ions are located on d sites, characterized by C_2_ (twofold rotational) symmetry. The oxygen ions are positioned on the e sites with C_1_ symmetry, indicating no symmetry elements (the most asymmetric site). This structure was drawn in Figure 9a. On the other hand, the monoclinic phase of Er_2_O_3_ belongs to the C2/m space group. Its unit cell is twice the size of the primitive cell, and contains 6 formula units, comprising a total of 12 Er^3^^+^ ions and 18 O^2^^−^ anions. The Er^3^^+^ ions are distributed across three different 4i Wyckoff sites (x, 0, z), all with relatively low symmetry. The oxygen ions occupy two types of sites 16 oxygen ions are on i sites with Cs (mirror plane) symmetry. The other two oxygen ions are located on b sites, which have C_2_h (twofold rotation + inversion) symmetry. This structure is illustrated in Figure 9b.

The bond length distributions of Er─Er in monoclinic and cubic Er_2_O_3_ are compared in Figure 9c. The average bond lengths of cubic Er_2_O_3_ and monoclinic Er_2_O_3_ were 5.50 ± 0.02 Å and 5.30 ± 0.05 Å, respectively. The monoclinic Er_2_O_3_ is found to have a wider bond length distribution and a shorter average bond length compared to cubic Er_2_O_3_. The relatively larger dispersion and interatomic distances of Er─Er bonds may result in a greater fraction of non‐radiative transitions, responsible for cross relaxation and energy loss. All these factors contribute to lower the PL efficiency of Er‐NPs composed of monoclinic Er_2_O_3_, and explain its lower value compared that of cubic Er_2_O_3_.

When the nearest coordination atom of the Er^3+^ ion is O, the smaller the coordination number of Er^3+^ ions and O, and the shorter the Er─O bond length, the greater the intensity of the energy transitions. The cubic phase is six‐fold coordination with O, while the monoclinic is seven‐fold coordination. The bond length distribution of Er─O monoclinic and cubic is presented in Figure 9d. It is shown that the Er─O bond length of cubic and monoclinic varies from 2.25 to 2.33 Å and from 2.17 to 3.93 Å, respectively, and the average bond length of cubic and monoclinic was 2.20 ± 0.08 Å and 2.70 ± 0.02 Å, respectively. As shown in Figure 9d and the statistical analysis results, the cubic phase has shorter Er─O bond lengths with a narrower length distribution compared to the monoclinic phase. Hence, similarly to Er─Er bonds, the reduction of Er─O interatomic distances in cubic Er‐NPs decreases energy barriers required for excitation energy transfer toward the Er^3+^ 4*f* emitting centers of Er‐NPs, which results in their more intense PL around 665 nm.

Such an interpretation of the PL measurements is further supported by the trends observed in the TGA showing the activation of critical structural rearrangements around 600°C, and XPS analyses conducted on the samples annealed at high temperatures. These latter show a reduction in Er_2_O_3_ content accompanied by the increase of structural disorder and the reappearance of erbium hydroxyl peaks after the sample cooled down, both of which are consistent with the re‐increase in interatomic distances reducing energy transfer mechanisms. Furthermore, the formation of excess Er─Er bonds or pure Er nano‐aggregates inside Er‐NPs above 800°C may introduce additional non‐radiative pathways that can exacerbate the decrease in PL intensity [65, 81, 82].

## 3 Conclusions and Perspectives

In this work, we investigated oxidized Er‐NPs synthesized via PLAL and subjected to post‐synthesis annealing at temperatures ranging from 200°C to 1000°C. To optimize their PL emission and ascertain the factors leading to variation in PL intensity (size, crystallinity, and the chemical changes induced by thermal treatments), we systematically examined their crystal structure and chemical composition after annealing. An increase in the oxidation state of Er, as well as a complete crystal transformation from a mixed monoclinic/cubic phase toward a pure cubic phase, is observed for Er‐NPs annealed at 600°C, where the maximal red PL emission is obtained. The variations in PL intensities are mainly attributed to the non‐radiative losses, associated with structural defects and disordering. The improvement in PL emission around 665 nm is driven by the significant reduction of the inter‐atomic Er─Er and Er─O distances, which result from the increase of oxygen ligands and crystal rearrangements leading to the significant volume compaction of Er‐NPs. This improvement in the optical performance of Er‐NPs highlights the preponderant role of excitation energy transfers for activating 4*f–*4*f* optical transitions. We believe that the implementation of this finding to Er‐based systems improves their general interest for telecommunication applications, and more particularly in optical amplifiers.

## 4 Experimental Section

### 4.1 Material Preparation

Er‐NPs were synthesized via PLAL using a 99.9% pure erbium metal target (The Kurt J. Lesker Co.), used as received. The ablation was performed with a Nd:YAG laser (Amplitude Technologies) operating at a wavelength of 532 nm, with a pulse energy of 50 mJ, a pulse duration of 12 ns, and a repetition rate of 10 Hz. The laser beam was focused perpendicularly onto the target surface using a concave lens with a 25 cm focal length, and the ablation process was maintained for 20 min. Full details of the NPs synthesis and solution preparation can be found in previous work [33].

For sample preparation, several drops of the colloidal Er‐NP solution were deposited onto silicon substrates and dried at 80°C in ambient air. Prior to deposition, all substrates were thoroughly cleaned and dried using nitrogen gas, and all colloidal suspensions were sonicated for 5 min to ensure proper dispersion. For thermogravimetric analysis (TGA), drops of the Er‐NPs solution were deposited onto platinum sample holders following the same cleaning and preparation steps.

The synthesized Er‐NPs were subsequently subjected to thermal annealing to evaluate the influence of temperature on their structural and optical properties. To prevent contamination from ambient gases, the samples were enclosed in a sealed quartz tube under a continuous flow of high‐purity nitrogen gas (grade 5.0). Thermal treatments were conducted at 200°C, 400°C, 600°C, 800°C, and 1000°C for 1 h. The annealing duration and nitrogen atmosphere were selected to minimize experimental variables and isolate the effect of temperature, which is known to be a critical parameter for crystal rearrangement. These conditions are comparable to those of previous studies aimed at determining the crystalline phase of Er‐based oxide systems [59, 83]. In this study, the dried non‐annealed samples are referred to as the “as‐deposited” for reference.

### 4.2 Material Characterization

XRD measurements were performed using an (X'Pert PANalytical) diffractometer equipped with a Cu K_α_ radiation source (λ  =  0.15406 nm), operated at 45 kV and 40 mA. A curved position‐sensitive detector was used to collect diffraction patterns within the *2θ* range of 20° to 70°.

The morphology and particle size of the Er‐NPs were analyzed using a Tescan LYRA 3 XMH SEM, coupled with energy‐dispersive X‐ray spectroscopy (EDS) from Bruker Nano GmbH (Esprit 2.0, Germany) for elemental analysis.

TGA was conducted using a TA Instruments Q500 system with a sensitivity of 0.1 µg and an accuracy of 0.01%. The temperature was precisely controlled up to 1000°C. Measurements were carried out in platinum (Pt) pans under Ar gas flow. The instrument was equipped with a quartz furnace.

Surface chemical composition and oxidation states were investigated using a PHI Quantes XPS apparatus equipped with a monochromatic Al K_α_ source (photon energy = 1486.6 eV). The analysis area was 0.4 mm in diameter with a probing depth of approximately 3–5 nm. Data processing and peak deconvolution were carried out using CasaXPS software (version 2.3).

PL spectra were recorded using a Horiba iHR320 spectrometer excited with a linearly polarized continuous‐wave diode‐pumped solid‐state (DPSS) laser (Cobolt 04‐01) at a 473 nm excitation wavelength. A x100 objective lens with a numerical aperture of 0.95 was used for focusing. Emission was detected using a 1024×256 pixel CCD camera (Horiba Synapse BIDD) through a 150 mm^−^^1^ diffraction grating, covering a spectral range from 475 to 900 nm. The exposure time varied between 30 and 120 s, depending on the signal intensity, with 2 to 4 acquisitions averaged to enhance the signal‐to‐noise ratio. To limit sample heating during PL measurements, all samples were placed on the same sample holder, exposed to room temperature, and the laser excitation power was kept around 10 mW. In order to verify that the samples were not damaged by the laser, we carefully examined their surface under an optical microscope to ensure that no visible changes were observed after each measurement.

Detailed morphological analysis was performed using a high‐resolution transmission electron microscope (JEOL JEM‐F200 Cold‐FEG TEM) operated at 200 kV, along with EDS for elemental analysis. TEM samples were prepared by dropping a colloidal Er‐NP solution onto carbon‐coated copper grids.

### 4.3 Statistical Analysis

Triplicate measurements (*n* = 3) were conducted for as‐deposited and thermally annealed samples at 400°C, 600°C, and 800°C. All experimental data, including XRD, XPS, SEM, and PL are reported as mean ± standard deviation (SD). Data processing and peak deconvolution for the XPS data were carried out using CasaXPS software (version 2.3).

Particle size distributions were quantified from SEM images by measuring at least 500 nanoparticles per sample using ImageJ software (NIH). The size data were fitted to log‐normal distribution functions to characterize the particle population. Crystal structure refinement and phase quantification were performed through Rietveld analysis of XRD patterns using Topas software. Interatomic distances (Er─O and Er─Er bond lengths) within the crystallographic unit cell were extracted from Crystallographic Information Files (CIF) and analyzed using OVITO visualization software to determine coordination environments. All statistical analyses, curve fitting, and data visualization were performed using OriginPro 2023.

## Conflicts of Interest

The authors declare no conflicts of interest.

## Supporting information




**Supporting File**: smll72963‐sup‐0001‐SuppMat.docx.

## Data Availability

The data that support the findings of this study are available from the corresponding author upon reasonable request.
