# Supplementary material for: Photoluminescence Enhancement in Erbium Nanoparticles via Controlled Phase Transformation
Source: Small. 2026 Mar 12;22(24):e11510. doi: 10.1002/smll.202511510 (PMC13114504; doi:10.1002/smll.202511510)
Supplement: Supplementary file 1 — Supporting File: smll72963‐sup‐0001‐SuppMat.docx. [file SMLL-22-e11510-s001.docx]

**Supporting Information**

**PHOTOLUMINESCENCE ENHANCEMENT IN ERBIUM NANOPARTICLES VIA CONTROLLED PHASE TRANSFORMATION**

B. Almohammed ^1,2^, D. Barba ^1^, E. Haddad ^3^, F. Rosei ^4^, F. Vetrone *^1, 2^

^1^ Centre Énergie Matériaux Télécommunications, Institut National de la Recherche Scientifique, Université du Québec, 1650 Boul. Lionel-Boulet, Varennes, Québec, J3X 1P7, Canada

^2^ Centre Énergie Matériaux Télécommunications, Institut National de la Recherche Scientifique, Campus Laval, Université du Québec, 531 Boul. des Prairies, Laval, Québec, H7V 1B7, Canada

^3^ MPB Communications Inc., 147 Boul. Hymus, Pointe-Claire, Québec, H9R 1E9, Canada

^4^ Department of Chemical and Pharmaceutical Sciences, University of Trieste, Via Giorgeri 1, Trieste, 34136, Italy

* Corresponding Author: Fiorenzo Vetrone (*fiorenzo.vetrone@inrs.ca*)


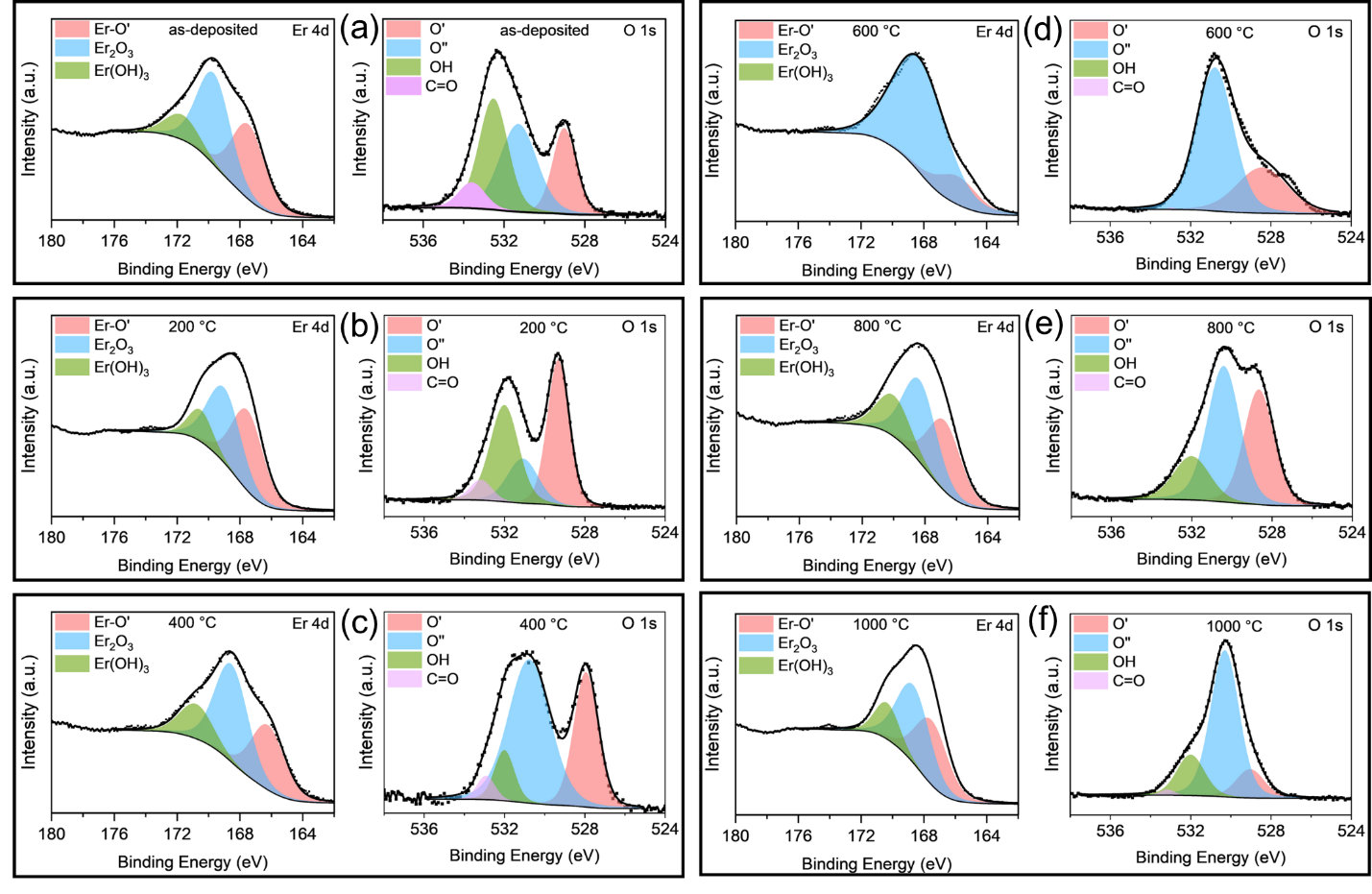


**Figure 1S:** Deconvoluted XPS spectra of the Er 4d (left) and O 1s (right) core levels for erbium nanoparticles annealed at different temperatures: (a) as-deposited, (b) 200 ^o^C, (c) 400 ^o^C, (d) 600 ^o^C, (e) 800 ^o^C, and (f) 1000 ^o^C. The fitted components correspond to Er-O’, Er_2_O_3_, and Er(OH)_3_ in the Er 4d region, and to O’, O’’, OH, and C=O species in the O 1s region.


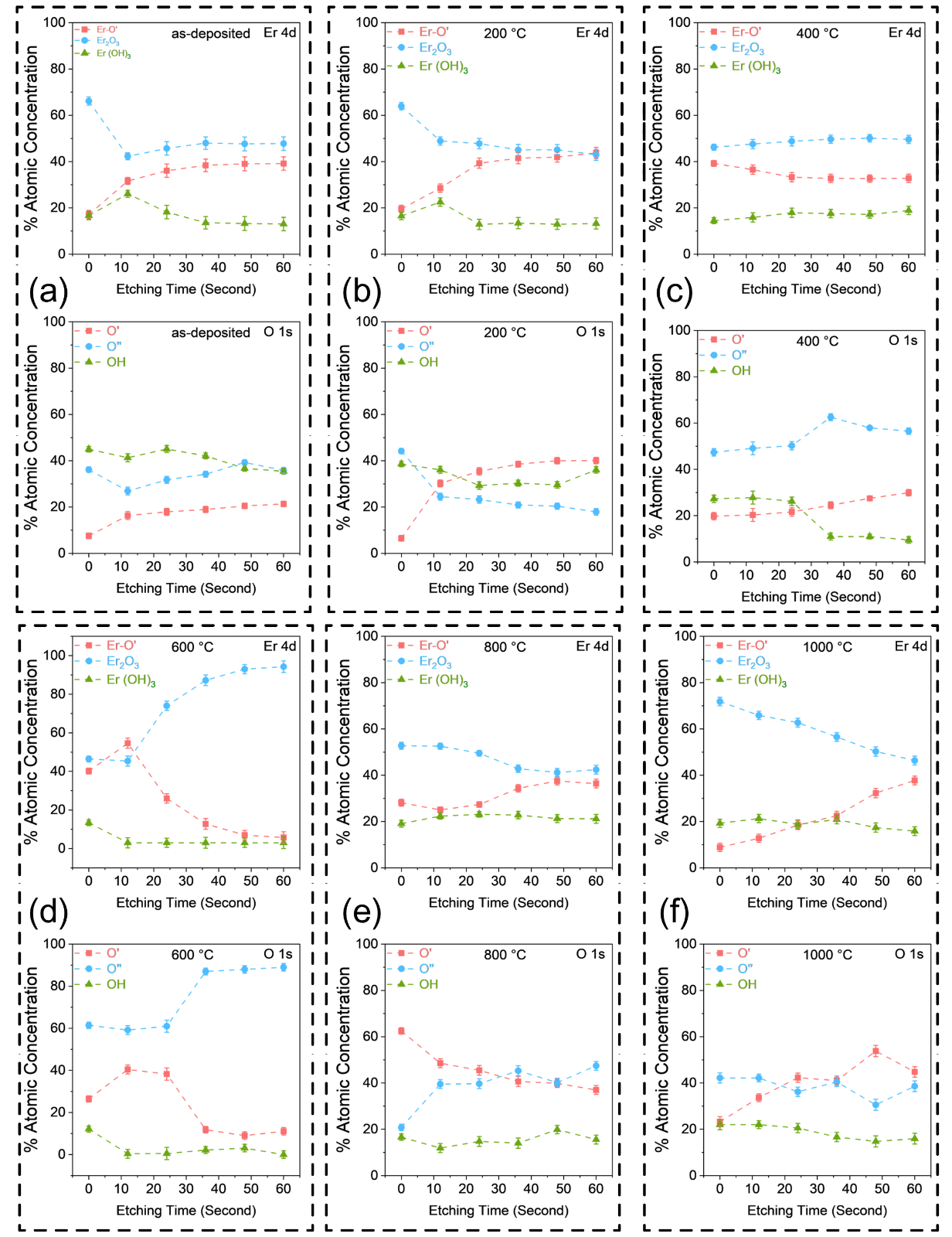


**Figure 2S.** Depth profile evolution of (top) Er 4d and (bottom) O 1s spectra for the as-deposited and annealed samples at 200 ^o^C, 400 ^o^C, 600 ^o^C, 800 ^o^C, and 1000 ^o^C. The plots show the variation in atomic concentrations of Er-O’, Er_2_O_3_, and Er(OH)_3_ species for Er 4d, and O’, O’’, and OH species for O 1s as a function of etching time, illustrating the compositional evolution with depth during thermal treatment.
